# My Wellbeing Journal: Development of a communication and goal‐setting tool to improve care for older adults with chronic conditions and multimorbidity

**DOI:** 10.1111/hex.13890

**Published:** 2023-10-13

**Authors:** Michael T. Lawless, Mandy M. Archibald, Rachel C. Ambagtsheer, Maria Alejandra Pinero de Plaza, Alison L. Kitson

**Affiliations:** ^1^ Caring Futures Institute, College of Nursing and Health Sciences Flinders University Bedford Park South Australia Australia; ^2^ Helen Glass Centre for Nursing College of Nursing Winnipeg Manitoba Canada; ^3^ Torrens University Australia Adelaide South Australia Australia

**Keywords:** aged, goal setting, multimorbidity, person‐centred care, shared decision‐making

## Abstract

**Background:**

Chronic conditions and multimorbidity, the presence of two or more chronic conditions, are increasingly common in older adults. Effective management of chronic conditions and multimorbidity in older adults requires a collaborative and person‐centred approach that considers the individual's goals, preferences and priorities. However, ensuring high‐quality personalised care for older adults with multimorbidity can be challenging due to the complexity of their care needs, limited time and a lack of patient preparation to discuss their personal goals and preferences with their healthcare team.

**Objective:**

To codesign a communication and goal‐setting tool, My Wellbeing Journal, to support personalised care planning for older adults with chronic conditions and multimorbidity.

**Design:**

We drew on an experience‐based codesign approach to develop My Wellbeing Journal. This article reports on the final end‐user feedback, which was collected via an online survey with older adults and their carers.

**Setting and Participants:**

Older adults with chronic conditions, multimorbidity and informal carers living in Australia. Personalised care planning was considered in the context of primary care.

**Results:**

A total of 88 participants completed the online survey. The survey focused on participants' feedback on the tool in terms of effectiveness, efficiency, satisfaction and errors encountered. This feedback resulted in modifications to My Wellbeing Journal, which can be used during clinical encounters to facilitate communication, goal setting and progress tracking.

**Discussion and Conclusions:**

Clinicians and carers can use the tool to guide discussions with older adults about their care planning and help them set realistic goals that are meaningful to them. The findings of this study could be used to inform the development of recommendations for healthcare providers to implement person‐centred, goal‐oriented care for older adults with chronic conditions and multimorbidity.

**Patient or Public Contribution:**

Older adults living with chronic conditions and multimorbidity and their carers have contributed to the development of a tool that has the potential to significantly enhance the experience of personalised care planning. Their direct involvement as collaborators has ensured that the tool is optimised to meet the standards of effectiveness and usability.

## 1 INTRODUCTION

Several countries are experiencing rapid and unprecedented population ageing, which has raised significant public health concerns regarding the growing prevalence of chronic conditions and multimorbidity.^1^, ^2^, ^3^ While several definitions exist, multimorbidity is typically characterised by the presence of two or more chronic conditions, and research indicates that multimorbidity affects over 50% of older individuals.^1^, ^2^ Furthermore, a recent meta‐analysis has indicated that 72% of those with multimorbidity also have frailty.^4^ Multimorbidity in older adults is associated with several negative consequences, including hospitalisation, functional decline, higher complexity of care and reduced quality of life.^5^, ^6^ The current approach to care for multimorbidity often focuses on the management of individual diseases, which can lead to polypharmacy, higher medication error rates, higher treatment burden (i.e., the workload associated with managing multiple treatments and health‐related recommendations and their impact on individuals and caregivers^6^), inattention to contextual factors and failure to align care with patients' goals, values and preferences.^7^, ^8^, ^9^ To effectively manage multimorbidity, healthcare providers must coordinate their efforts to ensure that care is not fragmented and is focused on what matters most to patients.^10^ This requires a shift towards a person‐centred approach that prioritises the individual's overall health and wellbeing, rather than just the management of single diseases.

Goal setting is a valuable strategy for promoting person‐centred communication and shared decision‐making, particularly among individuals with chronic conditions and multimorbidity.^11^, ^12^ The Ariadne principles, guiding the management of multimorbidity in primary care consultations, emphasise the importance of patients and healthcare providers establishing realistic goals.^13^ Goal setting involves a collaborative effort between patients, healthcare providers and carers. Together, they identify and agree on achievable goals, negotiate a plan to achieve those goals and subsequently assess goal attainment within a specified timeframe.^14^ Goal setting is a valuable strategy to enhance the care provided to older adults, aligning with the core principles of evidence‐based medicine and person‐centred care.^15^, ^16^, ^17^ These approaches aim to enhance care by incorporating patient values, preferences and the best available evidence. Given the complexity of multimorbidity and geriatric syndromes such as frailty, a multidisciplinary, team‐based approach to goal setting is often necessary.^18^ To facilitate integrated, person‐centred care, the healthcare team should ideally be multidisciplinary, including primary care physicians, nurses, physiotherapists, occupational therapists, psychologists, social workers and case managers, among other health and social care professionals. This collaborative approach ensures that patients receive comprehensive care that addresses their unique needs and preferences.^18^, ^19^, ^20^

Effective goal setting requires active engagement from both healthcare providers and patients. While the importance of healthcare provider preparation and training has been widely recognised, the specific modality, methods and duration of training vary among studies.^21^, ^22^, ^23^ Strong communication skills, including the ability to establish rapport, actively listen and engage in effective dialogue with patients, have been identified as key personal knowledge and skills for healthcare providers.^5^, ^15^, ^24^, ^25^ Additionally, studies have shown that older adults often feel unprepared to discuss their goals and preferences with healthcare providers.^15^, ^22^, ^23^, ^26^ To address this, a structured approach including appropriate tools might be implemented to facilitate goal setting and action planning for older adults in primary care settings.^27^, ^28^ These tools might be used to facilitate patient involvement in decision making, identify priorities, personalise care and support planning and track progress. Despite the growing interest in developing generic tools to support shared decision‐making, few have adopted a user‐centred, codesign approach involving older people and caregivers to optimise their relevance and effectiveness.^29^, ^30^, ^31^, ^32^ Therefore, it is crucial to involve older adults and their caregivers in the design and development of tools to ensure their effectiveness and relevance.

Research on patient and public involvement in healthcare service development is growing, with evidence suggesting positive impacts on research outcomes, policy, practice and service design.^33^, ^34^, ^35^ However, there are important ethical, methodological and practical challenges that need to be addressed when involving older adults as research partners.^36^, ^37^, ^38^, ^39^ James et al.^37^ found that codesign with older people can lead to various benefits including improved understanding of issues facing older people; more inclusive and responsive service design, practice and policy; ensure the acceptability of tools for older people; and opportunities for patient coresearchers to develop skills while giving a voice to marginalised groups of older people. Baldwin et al.^36^ found that involving older people in health and social care research can have benefits for older consumer coresearchers, academic researchers and research processes and outcomes. Moreover, Schilling et al.^39^ reported that effective involvement can be supported by building ‘good’ (e.g., equal) relationships, facilitating communication and breaking down barriers to participation such as providing a convenient and accessible location. While studies suggest that involving consumers in codesigning healthcare services is a promising approach, there is limited evidence on how to effectively involve older people with chronic conditions and multimorbidity in codesign processes. Markle‐Reid et al.^38^ identified five challenges and lessons for meaningfully engaging older adults with multimorbidity as research partners, including actively finding consumer partners who reflect the diversity of older people with multimorbidity, developing strong working relationships with patient partners and using flexible approaches for engaging patients.

The success of approaches like experience‐based codesign (EBCD) has demonstrated the potential of coproduction approaches and user‐centred design principles in enhancing patient care across various healthcare settings, including mental health, cancer care and emergency medicine.^40^ Understanding how and when to optimally engage older adults and their carers is central to ensuring meaningful involvement of older people in shaping healthcare services and ultimately improving their experiences and outcomes.^41^, ^42^, ^43^ In this study, EBCD was used to translate the available evidence and facilitate the development of a person‐centred tool to support goal setting and communication for older adults with chronic conditions and multimorbidity in primary care.

### 1.1 Goal setting and communication tool prototype version (booklet)

Recommendations to advance chronic disease and multimorbidity management for older adults in primary care include prioritising health problems, promoting shared decision‐making and setting realistic goals.^6^, ^8^, ^12^ In response to these recommendations, we have developed a novel communication and goal‐setting tool called My Wellbeing Journal to support person‐centred, goal‐oriented care for older people living with chronic conditions and multimorbidity. It was developed primarily for older adults with chronic conditions and multimorbidity, recognising that other populations might also find the tool useful. The tool was designed to help individuals discuss and keep track of their health and wellbeing goals and improve interactions between patients and their healthcare team. It is an A5 booklet divided into four sections: Exploring what matters; Doing what matters; Discussing what matters; and Journal entries. The journal format was chosen based on evidence for the interrelationship of narrative representation of experience and physical and mental health outcomes, as well as the ability to personalise self‐care support.^44^, ^45^, ^46^ The first section introduces the concept of goal setting and the various aspects of health and wellbeing that can affect individuals' goals. The second section provides further information about goal setting and tips for setting achievable goals based on specific, measurable, achievable, realistic/relevant, time‐bound (SMART) framework.^15^ The third section emphasises the importance of good communication between patients and their healthcare team and provides a list of prompt questions. The final section contains journal entries where users (with or without the assistance of a carer or healthcare provider, such as a primary care physicians/general practitioner, nurse, physiotherapist, occupational therapist or social worker) can record their overall quality of life, sources of stress, questions for their healthcare team, goals for the week/month and reflect on their progress.

We have collaborated with a range of end‐users, aligned with the principles of shared decision‐making and person‐centred care, to develop an evidence‐based tool with the potential to improve clinical care for older adults with chronic conditions and multimorbidity. Specifically, we wanted to ensure that the tool meets the needs of the intended users. The aims of this study were to: (1) codesign a communication and goal setting tool to support personalised care planning for older adults with chronic conditions and multimorbidity; and (2) explore lessons learned regarding how to involve these individuals in EBCD.

## 2 METHODS

**Figure 1:**
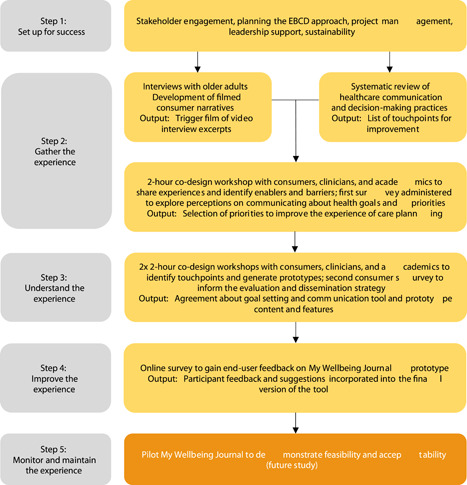
Overview of codesign process. EBCD, experience‐based codesign.

The development of My Wellbeing Journal was guided by the EBCD Australia Toolkit.^47^ EBCD is a narrative‐based, participatory approach that brings together professionals and service users to collaboratively design solutions. The EBCD toolkit provides a comprehensive five‐step approach to codesign that incorporates end‐users' experiences in a collaborative manner. The five steps are as follows: (1) set up for success; (2) gather the experience; (3) understand the experience; (4) improve the experience; and (5) monitor and maintain the experience. An overview of the codesign process is shown in Figure 1. Previous work has been conducted to complete Steps 1 through 3.^48^, ^49^, ^50^, ^51^, ^52^ The current study focuses on Step 4, which involves codesigning solutions based on end‐user feedback. Section 4.1 outlines the steps that will be taken for the fifth and final step, which involves monitoring the experience. Before reporting on the details and methods of Step 4, we provide background information on the previously completed steps. This information provides additional context about the development of the tool and how it was informed by end‐user experiences.

### 2.1 Steps 1–3: Tool development

Step 1, *set up for success* involves planning the EBCD approach and engaging with the stakeholders who will be impacted by the project. Between January and March 2021, two authors (M. L. and M. A. P. P.) recruited consumer partners to join the codesign group through a formal position description and call for expressions of interest, targeting individuals with experience as an older adult (over age 65) living with chronic health condition(s) or in a caring role for an older person with a chronic condition(s). We also invited academics and clinicians affiliated with three South Australian universities to participate in the codesign group. Additionally, we established an online consultation cohort of consumers (*n* = 10). M. L. engaged an external creative facilitator to support all team members during the codesign process. Before the study commenced, M. L. communicated with the facilitator on several occasions to establish the objectives and parameters of the project, create workshop materials and explore techniques and resources to facilitate effective collaboration and innovative ideation.

Step 2, *gather the experience*, involves gaining an understanding of the current experience to inform the development of potential solutions. We began by triangulating the findings of a large qualitative study^48^, ^49^ and a systematic review,^51^ which are reported elsewhere. The qualitative study aimed to understand the experiences and perceptions of various healthcare provider groups and consumers about frailty and frailty screening. As part of this study, we conducted seven focus groups with older adults (*n* = 39) aged 62–99 years (*M* = 80.6, SD = 9.6) from community, assisted living and residential care settings,^48^, ^49^ and produced a series of edited videos that reflected central themes and priorities from the consumer experience interviews that could be used to stimulate future codesign work.^50^ Additionally, we conducted a literature review of observational research on communication between healthcare providers, older adults and carers about self‐management goals and actions.^51^

Following this, we conducted three in‐person codesign workshops in Adelaide, South Australia.^52^ The codesign group consisted of academics and clinicians (*n* = 7) and consumers (*n* = 3). This sample size corresponds to the ‘small codesign team’ stage reported in previous studies using EBCD.^53^ Participants were selected for their diverse expertise and qualifications, encompassing lived experience, clinical practice, research, public service, consumer advocacy and creative facilitation. This ensured that the codesign group had a comprehensive, multidisciplinary perspective and the required expertise to develop effective solutions for this population.

The codesign workshops aimed to generate improvement ideas and refine prototypes. All participants gave informed consent for audio recordings to be used for research purposes. At the beginning of the workshop (March 2021), a prerecorded video (‘trigger film’) was played to stimulate discussion using authentic consumer narratives from our previous qualitative research. The first session's main task was to identify enablers and barriers to older adults' participation in healthcare discussions about their goals and share improvement ideas. Building upon the insights gained during the first workshop, we conducted an initial survey among the online cohort. The first survey aimed to assess the importance of various topics in goal‐setting discussions between patients and their healthcare providers, gather insights into factors that support or hinder older people's participation in such discussions, and explore potential actions individuals might take as a result of discussing their goals with healthcare professionals.

Step 3, *understand the experience*, involves taking knowledge from previously completed steps to stimulate further discussion and creative ideation. During the second workshop (April 2021), the codesign group completed a prototype mapping activity to identify key moments in the consumer and healthcare provider journeys, propose solutions, and develop a range of potential prototypes. The third workshop (May 2021) focused on reviewing prototypes, observing group reactions and discussing modifications and refinements. The online cohort completed a second survey conducted in June 2021. The survey sought insights into how individuals search for health information online, the websites they commonly use, and key design factors to consider when creating health resources for patients and caregivers. The findings from this survey provided information to revise the prototype and inform the evaluation and dissemination strategy. The codesign group maintained regular communication, engaging in feedback and revision cycles from June to September 2021. The feedback received during this period was incorporated into the tool's further development.

**Figure 2:**
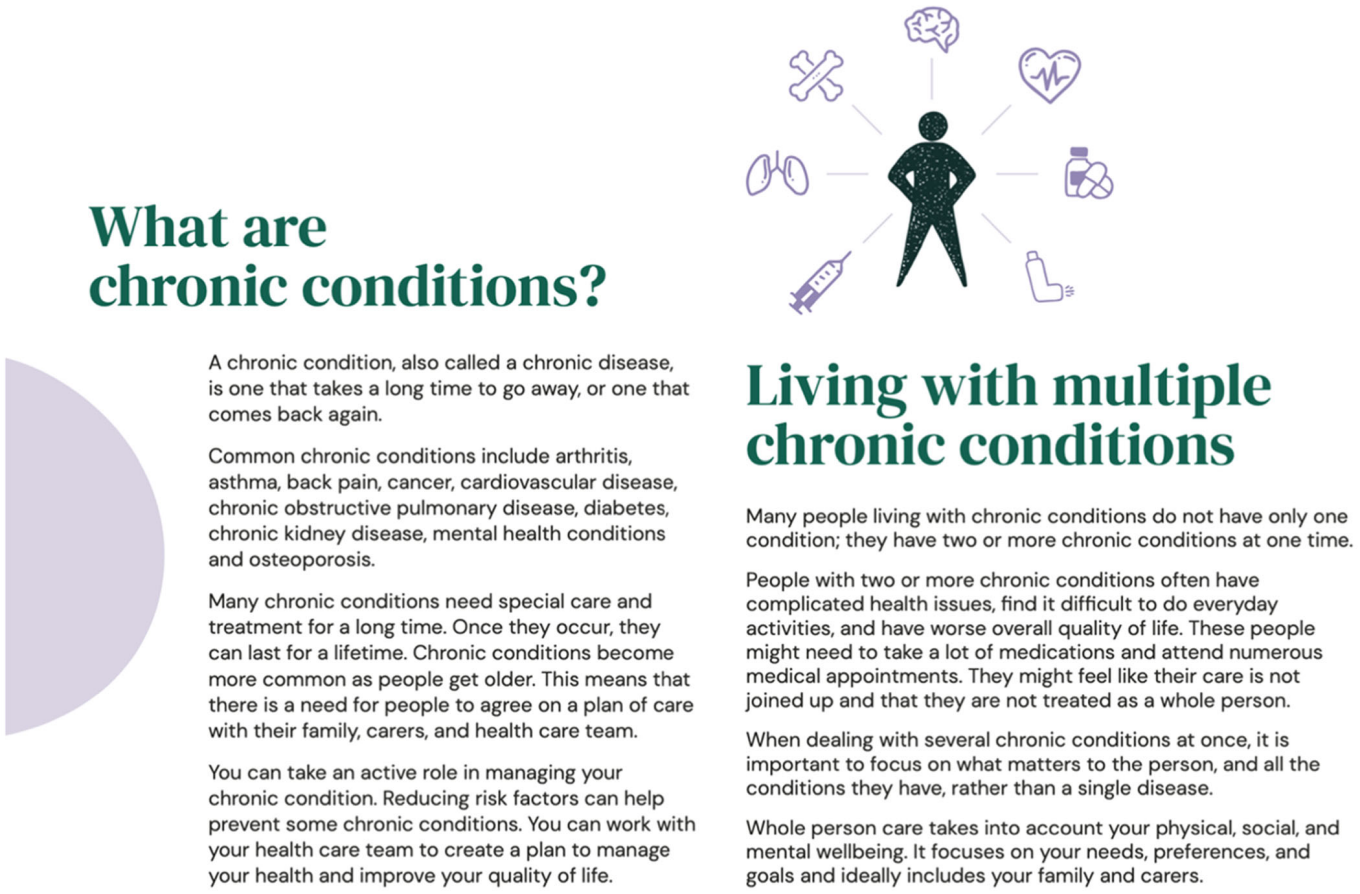
Example page from ‘My Wellbeing Journal’ (Step 1: Exploring what matters).

**Figure 3:**
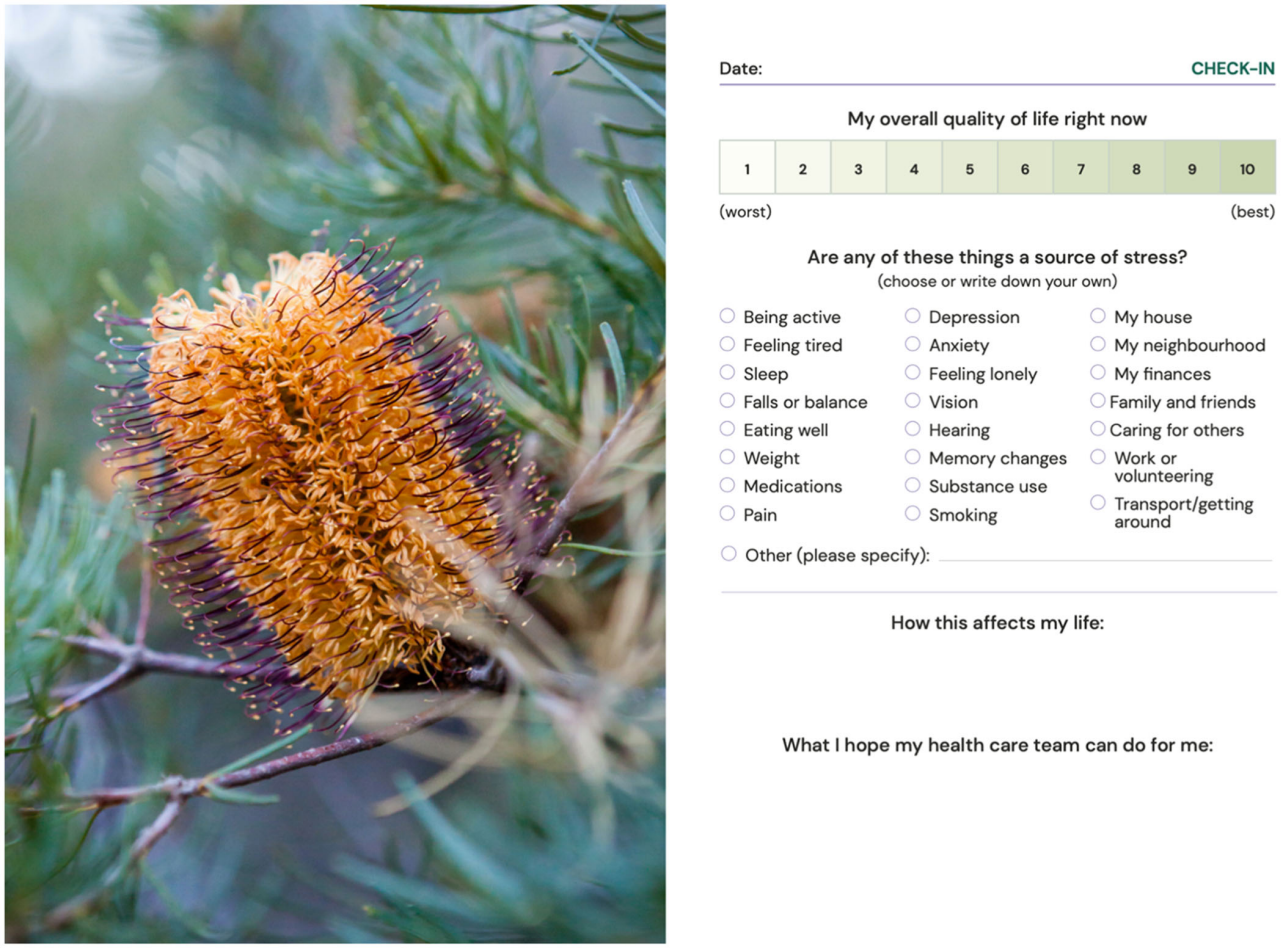
Example page from ‘My Wellbeing Journal’ (Journal check‐in).

**Figure 4:**
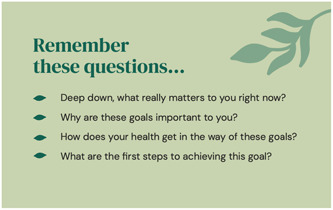
Prompt card with example questions.

The information gathered from the workshops, qualitative study and systematic review were used to produce a high‐fidelity prototype, based on the agreed specifications, as shown in Figures 2, 3, 4. Accessibility was assessed using the Web Content Accessibility Guidelines 2.0 Level AA (acceptable compliance) as a minimum, meaning that the prototype is usable for most people with and without disabilities. We also used the Simple Measure of Gobbledygook readability index,^54^ equivalent to a Grade 8 standard of reading wherever possible, which is the recommended standard for health‐related information in Australia.^55^ Based on recommendations, we modified the content and developed a prototype goal setting and communication tool (booklet) for pilot testing. The development of the tool followed the International Patient Decision Aid Standards collaboration guidelines.^56^

### 2.2 Step 4: End‐user feedback

Step 4, *improve the experience*, involves translating the understanding of the experience into meaningful improvements. Before making the tool available for use, we wanted to give end‐users the opportunity to provide feedback on the prototype version and provide survey‐based feedback and comments. This feedback was collected and reported in the current manuscript. To gain feedback from participants, we used convenience sampling to recruit older adults and carers using the consumer mailing lists of a national consumer advocacy organisation working with carers and older people. All participants were invited to review a web‐based version of My Wellbeing Journal from their computer or mobile device and complete an online survey using Qualtrics XM (Qualtrics International Inc.). The survey, inclusive of reviewing the tool, was designed to take 30 min to complete. The survey included demographic questions, Likert items assessing level of knowledge, confidence in setting goals and managing health conditions day‐to‐day, and specific questions related to the tool's usability (e.g., is it useful) and acceptability (e.g., is it visually appealing), as well as open‐ended questions asking participants to provide their positive and negative opinions. Likert items ranged from 1 (*not at all*) to 10 (*very much*). Data were collected in May 2022. Ethical approval was provided by the Flinders University Human Research Ethics Committee (project number 5254). All participants provided informed consent before taking part in the survey.

### 2.3 Data analysis

Categorical data were analysed using descriptive statistics, whereas open‐ended survey responses were analysed inductively using conventional qualitative content analysis.^57^ To ensure the accuracy and reliability of the qualitative data analysis, M. L. initially grouped the responses into categories. The categorisations were then reviewed and refined by M. A. P. P., M. A., R. A. and A. K., and were finalised through group consensus. The team also employed detailed analytic memos and held regular meetings to enhance the rigour of the analysis. The findings for each category were summarised in brief narrative descriptions.

## 3 RESULTS

A total of 107 participants accessed the survey and 88 participants returned a completed survey. Characteristics of the survey respondents are shown in Table 1. Most of the participants were aged over 65 years (*n* = 80, 90.91%), male (*n* = 55, 62.50%); the mean age was 65.57 years (SD = 8.72). The most common self‐reported chronic conditions were arthritis (*n* = 20, 18.35%), diabetes (*n* = 19, 17.43%) and asthma (*n* = 16, 14.68%). Fourteen participants (15.9%) had two or more chronic conditions. All participants spoke English as the main language at home. Descriptive statistics indicated positive ratings on constructs related to usability (Table 2).

**Table 1:** Demographic characteristics (*n* = 88).

Characteristic	*N* (%)
Aged 65 years or above	80 (90.91)
Carer^a^	8 (9.09)
Age in years (mean, SD)	65.57 (8.72)
Gender	
Male	55 (62.50)
Female	33 (37.50)
Nonbinary	0 (0)
Chronic condition diagnosis	
Arthritis	20 (18.35)
Asthma	16 (14.68)
Cancer	8 (7.34)
Dementia	3 (2.75)
Diabetes	19 (17.43)
Heart disease	11 (10.09)
Kidney disease	5 (4.59)
Lung condition	10 (9.17)
Mental health condition	6 (5.50)
Stroke	10 (9.17)
Other	1 (0.92)
Total number of chronic conditions	1.23 (0.60)
Number of visits to healthcare provider in past year	
12+	7 (7.95)
7–12	20 (22.73)
4–6	29 (32.95)
1–3	32 (36.36)
0	0 (0)
Australian state/territory	
Australian Capital Territory	7 (7.95)
New South Wales	16 (18.18)
Northern Territory	0 (0)
Queensland	19 (21.59)
South Australia	19 (21.59)
Tasmania	1 (1.14)
Victoria	15 (17.05)
Western Australia	11 (12.50)
Location^b^	
Urban location (e.g., city or metropolitan area)	68 (77.27)
Country, rural or remote	20 (22.73)
Main language spoken at home	
English	88 (100)
Highest level of education	
Postgraduate university	6 (6.82)
Undergraduate university	64 (72.73)
Vocational training (e.g., technical and further education)	6 (6.82)
Completed high school	10 (11.36)
Completed some high school	2 (2.27)

^a^ Carers were aged under 65 years and provided unpaid care, help or assistance to a family member or others because of disability, long‐term health conditions or older age.

^b^ Location classified according to Australian Bureau of Statistics remoteness structure: Locality to remoteness area concordance.

**Table 2:** Descriptive statistics of usability findings (*n* = 88).

Item	Mean	SD	Range
Effectiveness			
It is useful	7.50	1.82	4–10
It provides information that is relevant to me	7.25	2.08	2–10
It helps me make decisions about my health	7.47	1.70	4–10
It is useful to share with my doctor	7.43	1.76	2–10
Efficiency			
It is simple to use	7.48	1.86	2–10
I can use it without assistance	7.35	1.91	3–10
The length is appropriate	7.57	1.73	4–10
Satisfaction			
The tone and feel are appropriate	7.15	1.93	2–10
It is visually appealing (e.g., images, colours)	7.38	1.90	3–10
I would use it in the future	7.56	1.79	3–10
I would recommend it to a friend	7.63	1.72	2–10

### 3.1 The contents of the journal are informative and relevant

Participants commented that the information contained in the Journal was useful (*M* = 7.50) and relevant to them (*M* = 7.25), as demonstrated by the quantitative ratings. Open‐ended responses suggested that many participants found the contents of the Journal useful (e.g., ‘[My Wellbeing Journal] contains a lot of information that I want and can just apply to my life’). Participants had positive reactions to the care planning and goal setting aspects of the journal, as well as the information about how to communicate effectively with the healthcare team. As one participant commented, ‘I loved the ability to be able to write down my goals and the prompt to record whether I had achieved them’. Another participant stated that the information contained in the journal is ‘especially helpful for the elderly’. In general, participants felt that the journal helped them make decisions about their health and care, stating that ‘it is a sound basis for helping people with their health problems’, reflected in quantitative ratings (*M* = 7.47). Furthermore, participants were satisfied that the journal contains information that is useful to share with their healthcare provider (*M* = 7.43).

### 3.2 The journal format is simple and convenient

Participants indicated that the tool is convenient and simple to use (*M* = 7.48), supported by two open‐ended responses (e.g., ‘simple and convenient to use’). Most participants were indicated that they were able to use the journal without assistance from others (*M* = 7.35). On average, participants reported that it took 15 min and 34 s (SD = 5.92) to read the journal, which was regarded as an appropriate length (*M* = 7.57).

### 3.3 The design of the journal is appealing and appropriate

Participants expressed that the language and visual aesthetic of the journal is appropriate and the tone and feel of the journal were generally rated positively (*M* = 7.15). Participants commented that the journal is calming, personal and encouraging, such as ‘it gives me something positive to look forward to’. Similarly, participants reported that the journal is visually appealing (*M* = 7.38). When compared to standard information (e.g., brochures, checklists) provided by healthcare providers, the journal was found to be preferable (e.g., ‘[My Wellbeing Journal is] far superior to much that I've seen’). Participants reported that they would use the journal in the future (*M* = 7.56, intention to use measure) and that they would recommend it to a friend (*M* = 7.63, likelihood to recommend measure). Overall, the qualitative findings support and elaborate on the quantitative results: participants found the journal accomplished the task of preparing older people with chronic conditions and their informal carers to discuss and keep a record of their health and wellbeing goals.

### 3.4 Negative feedback and revision of the journal

Two participants commented that some of the language was academic rather than focused on the target audience of older people and their carers (e.g., ‘the difficulty with all of these things I think is that trying to translate from a theoretical academic view … down to an old person who hasn't got a clue where you're talking about’). Second, one participant commented that there was no provision to keep monthly records of success and failure. Two additional open‐ended comment suggested that some of the colours were too bright and might be unsuitable for people with visual impairments (e.g., colour blindness). The feedback regarding these issues, as well as any other problems or errors, was shared with the development team during the subsequent revision of the Journal (Version 2).

## 4 DISCUSSION AND CONCLUSION

### 4.1 Discussion

My Wellbeing Journal is a user‐centred and evidence‐based tool that has the potential to support personalised care planning for older adults living with chronic conditions and multimorbidity. To ensure that the tool meets end‐users' needs, its development was conducted in four steps, consistent with the first four steps of the five‐step EBCD Australia Toolkit.^47^ The current study reported specifically on Step 4 and analysed the feedback and comments from 88 participants who tested the tool and completed an online usability questionnaire. Based on this feedback, we have incorporated all feasible changes (e.g., clarity of language, colours and fonts) into the final version of the tool (Supporting Information: Appendix S1). Overall, the findings indicate that My Wellbeing Journal is a helpful tool for preparing older adults with chronic conditions and their carers to discuss and keep a record of their health and wellbeing goals. Considerations for those who intend to develop creative knowledge translation tools with older individuals in different contexts are summarised in Table 3.

**Table 3:** Summary of design and usability considerations and key findings.

Decision point	Preference	Justification and considerations
Colour palette	Natural tones Accessible	Soothing colour psychology and calming nature visual identities (i.e., brand message). Dark base colours used with light colours are all accessible following AA (mid‐range) accessibility standards at a minimum (Web Content Accessibility Guidelines). Some colours are only accessible when used at a larger size.
Text font and size	Easy to read Digitally compatible	Low contrast geometric sans serif font for use at smaller text sizes (body and subheadings, e.g., DM Sans, Georgia, Arial). Font is not available in some digital environments (e.g., marketing automation platforms such as MailChimp have limited font libraries as do email browsers and digital signatures).
Language and tone	Plain English	Suitable for people with low literacy or English as a second language. Use of wording that promotes understanding and avoids ageist language.
Images and iconography	Context‐specific	Images of natural scenes strong associations with Australian context and journal branding. Avoid using icons in place of text as some users might be unfamiliar with their meaning.
Structure	Four sections with journal entries Link to supporting information	Simple and convenient to use to promote comprehension, relatability and acceptability. Use of summaries, transitional text and organised into sections as appropriate. All references, articles and websites are accessible via a QR code to improve relatability avoid overly academic appearance.
Time to complete	<15 min	Appropriate length for integration into routine self‐care activities. Individual journal entries can be completed separately.
Delivery method	Digital and hard copy	Hard copy version regarded as convenient and suitable for people with limited digital literacy. Digital version regarded as contemporary, interactive and accessible.

My Wellbeing Journal can be implemented in various ways, including self‐guided use, enabling patients use the tool independently, which could be made available online, as a mobile app or as a paper‐based journal. Alternatively, it can be used with the support of carers and family members who can assist older adults in setting goals and sharing journal entries with healthcare providers. Healthcare providers could introduce My Wellbeing Journal as part of routine care for older people with multimorbidity to structure the initial consultation and subsequent interactions. During appointments, the tool can facilitate communication, goal setting and progress tracking. Healthcare providers can also review journal entries during follow‐up appointments to ensure that patients' goals are being met. Implementing the tool into practice will require a multifaceted approach that addresses the barriers to adoption and ensures successful integration with existing workflows and systems. Although this study suggests that the tool requires a relatively low time investment, this finding should be viewed considering potential variations related to extended communication or the integration of additional communication or decision support tools during consultations, as this could impact adoption. To successfully implement the tool, healthcare providers and staff will need training and champions need to promote adoption and appropriate use of the tool. The tool should be tailored to local contexts and integrated with existing electronic health records and/or patient portals to facilitate access and use.^29^, ^58^ Regular monitoring and evaluation of the tool's use can help identify barriers and opportunities for improvement. Patient feedback and usage data could guide refinements and optimise the tool's impact.

In considering the implementation of My Wellbeing Journal, it is valuable to compare it with existing tools, such as the Instrument for Patient Capacity Assessment (ICAN) Discussion Aid.^29^ ICAN, developed using a user‐centred design approach, facilitates discussions between clinicians and patients regarding treatment burden and the patient's capacity to enact treatment work, aligned with the principles of minimally disruptive medicine.^59^ Like My Wellbeing Journal, ICAN is designed to be flexible rather than prescriptive, allowing patients and healthcare providers to collaboratively engage in problem‐solving around the identified issues. In the pilot evaluation of ICAN, primary care encounters involved observably different conversation topics from usual care conversations, particularly in terms of discussing lifestyle (e.g., diet and physical activity) recommendations, medication adherence and competing priorities.^60^ ICAN was perceived as feasible for use in primary care without extending the duration of visits. While both tools show promise for improving patient care, their successful integration into clinical practice requires a comprehensive understanding of implementation barriers, training needs and adaptation to local healthcare contexts.

To complete Step 5 of the EBCD toolkit, *monitor and maintain the experience*, and ensure that end‐users can access and provide feedback on My Wellbeing Journal, it has been made publicly available online (https://issuu.com/mywellbeingjournal/docs/flinders_wellbeing_journal_fa_web). Data on online engagement with the tool will be monitored to inform future evaluation activities and revisions. The next phase of this research programme will involve pilot testing and evaluating the feasibility of using My Wellbeing Journal in primary care consultations. This research will provide valuable insight into whether using My Wellbeing Journal in primary care is efficient, feasible and capable of modifying goal‐setting conversations with older people and carers. The research will also evaluate clinician‐ and patient‐perceived success of consultations, length of consultations and topics of discussion. Lessons learnt in this pilot will inform a larger randomised controlled trial.

Our research highlights the advantages of adopting a user‐centred codesign approach when creating patient decision aids and other personal health tools through the combination of EBCD and usability testing concepts.^61^ Most participants in our study expressed their satisfaction with the tool's aesthetics and research‐based content and indicated their willingness to use it in the future. By involving older adults and clinicians in advisory and partnership roles throughout the development process, we were able to enhance the relevance, credibility and aesthetic appeal of the tool while adhering to established usability principles. However, codesign can present challenges when balancing aesthetic and usability aspects with the integrity of research data.^62^, ^63^ For instance, Le et al.^64^ discussed the difficulties of creating an arts‐based knowledge translation tool for paediatric procedural pain that aligned with parents' preferences for aesthetics, usability, ease‐of‐use and length. Despite these challenges, other authors have found that iterative codesign approaches are beneficial for maximising acceptability, usability and enjoyability over time, particularly when developing creative knowledge translation tools, although such approaches are rarely used in practice.^65^

An aim of this study was to explore lessons regarding how to involve older adults with chronic conditions and multimorbidity in EBCD. A challenge in this study related to recruiting consumer partners in EBCD that reflected the diversity of older people with chronic conditions and multimorbidity using conventional recruitment channels (e.g., consumer mailing lists, newsletters), as highlighted in previous studies.^38^ This includes considering factors such as sexual and gender identity, socioeconomic status, cultural background and the specific chronic conditions experienced. We acknowledge the value of applying diversity, equity and inclusion principles in future EBCD work to ensure representation of hard‐to‐reach and underrepresented subgroups of older adults. Building rapport and trust by investing time in developing relationships with consumer partners and defining clear expectations for consumer roles and responsibilities, was essential to maintaining engagement and ongoing communication across the different stages of EBCD. Additionally, we learned the importance of addressing practical and logistical challenges such as transportation, accessibility and time commitment by providing accessible meeting locations and transportation assistance if required.^39^ We were conscious of the need to balance power dynamics and facilitate participation by fostering an environment where all participants feel empowered to contribute their experiences and insights. Although we strived to ensure equal participation, we cannot claim that we overcame the power imbalance that may exist or be perceived to exist between researchers, professionals and consumer partners.^66^ We used various documented strategies to attempt to equalise this power imbalance, including providing financial compensation for consumer partners' time and expertise at all stages, sharing information and decision‐making power with consumer partners (e.g., involving consumers in prototyping and user testing; using voting, prioritisation and scenario‐based decision‐making; appointing consumer coresearchers), and involving an external facilitator to ensure equal participation of consumers during meetings.^38^

Some limitations of our study should be noted. First, the demographics of this study and the convenience sampling approach, while efficient to implement, limit the generalisability of our findings. The participants were, on average, younger and reported a high level of education and fewer comorbidities, which might not be representative of older people with multimorbidity in primary care with varying levels of education, health literacy and cognitive function.^10^ All participants were English speakers, with the majority being men, and a small number of carers were included. These demographic characteristics may limit the applicability of our findings to primary care populations. It is possible that individuals who did not respond to the survey would have provided different perspectives. Consequently, further work is needed to ensure the relevance of the My Wellbeing Journal to consumers and healthcare providers and examine the utility of the tool as part of personalised care planning interventions involving collaborative goal setting and action planning and/or comprehensive self‐care toolkits tailored to the specific needs of older adults. Furthermore, future research could involve direct observation of users, such as think aloud methods to ensure that interviews included specific questions about how users interacted with the prototype in a naturalistic environment^62^ for example, in primary care encounters.^60^ This would likely have identified different considerations relevant to clinical encounters in primary care. Lastly, while the engagement process was not formally evaluated in this study, it is advisable for future research to assess the feasibility and acceptability of the codesign process among both patients and clinicians.

### 4.2 Conclusion

My Wellbeing Journal is a promising tool that offers a structured approach to discussing and tracking older adults' personal health and wellbeing goals. By prioritising these goals in alignment with their care, individuals can facilitate conversations with caregivers and healthcare professionals about their needs, goals and priorities. This tool has the potential to support proactive, personalised care and support planning for older people by guiding conversations between patients and healthcare providers to clarify goals, options, preferences and agree on a plan of action. Further research will be conducted to evaluate the feasibility of using My Wellbeing Journal in primary care and its impact on clinical care. In addition, a codesign process with healthcare providers will be used to develop and test education and communication training materials to support personalised care planning for older people with chronic conditions and multimorbidity.

## CONFLICT OF INTEREST STATEMENT

The authors declare no conflict of interest.

## ETHICS STATEMENT

This project obtained ethical approval from the Flinders Social and Behavioural Ethical Committee (project number 5254).

## Supporting information

Supporting information.

## Data Availability

The data that support the findings of this study are available from the corresponding author upon reasonable request.
